# When There Are a Myriad of Causes, and It's the Rarest One: A Case Report of Mediastinal Teratoma

**DOI:** 10.7759/cureus.102653

**Published:** 2026-01-30

**Authors:** Afonso H Cardal

**Affiliations:** 1 Family Medicine, USF (Unidade de Saúde Familiar) da Baixa, ULS S. José (Unidade Local de Saúde de São José), Lisbon, PRT

**Keywords:** adenopathy, anterior mediastinal mass, chest pain, mediastinal teratoma, odynophagia

## Abstract

Lymphadenopathy and chest pain are frequent reasons for consultation in primary health care, with a myriad of possible etiologies, ranging from infections to malignancy. When these symptoms persist or present with warning signs, longitudinal reassessment, progressive integration of clinical findings, and appropriate investigation become essential for diagnosis. A case is presented of a 40-year-old man of Nepalese origin who was first seen in 2015 for cervical lymphadenopathy associated with odynophagia in the context of tonsillitis. Despite partial improvement after adequate treatment, the patient maintained lateral cervical and supraclavicular lymphadenopathies and did not return for follow-up. He returned in 2017 due to persistent lymphadenopathies and occasional episodes of retrosternal pain and a feeling of pressure in the lower throat, losing follow-up once again afterward. Between 2021 and 2023, he continued to have similar episodes, developing chest pain with pleuritic characteristics. Several etiologies were considered, and the corresponding tests and treatments were performed, without relevant changes or symptom remission. In December 2023, the patient underwent a thoraco-abdominopelvic computed tomography (CT) scan that revealed an anterior mediastinal mass with adjacent lymphadenopathy, raising concern for malignancy. He was urgently referred to hospital care and underwent a biopsy that revealed a mature teratoma. In 2024, surgical excision was performed via video-assisted thoracoscopic surgery (VATS), revealing adhesion to the pericardium and proximity to the phrenic nerve and brachiocephalic venous trunk. The patient progressed with complete remission of symptoms. This case highlights the importance and possible complexity of continuity of care, of the assessment of persistent signs and symptoms, and of the temporal integration of clinical findings, which are fundamental for the identification of rare diagnoses, such as mediastinal teratoma, even in situations of low adherence to the care plan.

## Introduction

Peripheral lymphadenopathy and chest pain are common complaints in primary health care and frequent reasons for consultation. In most cases, they have a benign and self-limiting origin associated with infectious processes. However, they may represent manifestations of systemic, autoimmune, or neoplastic diseases, or even be iatrogenic [1]. The challenge for the family physician lies in identifying and distinguishing self-limiting situations from those requiring in-depth investigation or prolonged surveillance. For this, a flexible and comprehensive approach is necessary, ranging from active surveillance with anamnesis, physical examination, and reassessment to directed complementary investigation according to symptom persistence or the existence of warning signs, which is fundamental in the context of family medicine.

In the evaluation of lymphadenopathy, some findings should serve as warning signs for additional investigation. Among the criteria that justify this investigation are duration longer than four weeks, diameter greater than 1.5-2 cm, hardened consistency or fixed presentation, some specific locations, and the presence of systemic symptoms such as fever, night sweats, or concomitant weight loss [2].

Chest pain, in turn, can arise from multiple etiologies, the most common being cardiovascular, pulmonary, gastrointestinal, musculoskeletal, and psychogenic causes. Although most causes are benign, persistence, atypical presentation, or association with warning signs should motivate reassessment and, when indicated, complementary examinations [3,4].

Mediastinal teratomas are rare germ cell tumors, representing approximately 5% to 15% of mediastinal masses in adults, typically located in the anterior mediastinum [5]. The diagnosis is sometimes incidental, but compressive symptoms, such as chest pain, cough, dysphagia, or dyspnea, may arise, motivating investigation. Surgical treatment generally has a good prognosis when excision is complete [6,7].

This case report describes the prolonged and atypical evolution of a patient initially evaluated for lymphadenopathy and odynophagia, who later developed chest pain with variable characteristics. The final diagnosis of mature mediastinal teratoma, after eight years of the initial complaints, highlights the importance of valuing persistent signs, continuous reassessment, and integration of information over time--fundamental principles of family medicine.

## Case presentation

A 40-year-old man of Nepalese origin, working in the restaurant industry, with no known relevant personal history, and an occasional smoker, presented with cervical lymphadenopathy associated with odynophagia in the context of tonsillitis. He had sporadic contacts with his primary healthcare center over time, being seen by multiple healthcare professionals in same-day consultations (unscheduled acute care visit) and only occasionally by his assigned family physician in scheduled appointments. The patient’s clinical course, investigations, and outcomes over the eight-year follow-up period are summarized in Table 1.

**Table 1:** Timeline of clinical findings, investigations, and outcomes

Time Period	Clinical Presentation	Investigations Performed	Key Findings	Outcome
Aug-Sep 2015	Left cervical lymphadenopathy (2 cm), odynophagia, weight loss (6 kg/month)	Laboratory tests, cervical ultrasound	Submandibular and left supraclavicular lymphadenopathies with reactive characteristics	Biopsy declined; patient lost to follow-up
2017-2020	Persistent cervical and axillary lymphadenopathies, intermittent retrosternal pain, sensation of pressure in the lower throat	ECG, laboratory tests, ultrasound	Normal ECG; no significant changes on ultrasound	Sporadic follow-up with multiple healthcare professionals
Aug 2021	Right-sided pleuritic chest pain, previous episodes of precordial pain	Chest radiography, ECG	Both unremarkable; local tenderness on palpation	Musculoskeletal etiology considered; NSAIDs prescribed; missed follow-up
Jul-Oct 2023	Pharyngeal discomfort (“stuck fish bone” sensation, heartbeat sensation in the lower throat), stabbing chest pain (minutes to hours), aggravated by deep inspiration	Holter monitoring, echocardiogram	Both unremarkable	Nonsteroidal anti-inflammatory drugs (NSAIDs) and proton-pump inhibitors (PPIs) with partial improvement; sent to the emergency department, discharged without a clear diagnosis
Nov 2023	Symptom worsening	Thoraco-abdominopelvic CT scan	Anterior mediastinal mass (54×49×47 mm) with central necrotic areas; adjacent lymphadenopathy (9 mm)	Urgent referral to hospital care for suspected malignancy
Dec 2023	-	Transthoracic needle aspiration biopsy (TNAB)	Compatible with mature teratoma	Definitive diagnosis established
Feb 2024	-	Video-assisted thoracoscopic surgery (VATS)	6 cm hard mass adherent to pericardium, related to right phrenic nerve and brachiocephalic venous trunk; pathology: mature cystic teratoma with necrosis, free margins	Complete surgical excision; complete symptom resolution

August to September 2015

The first observation in the emergency department was for left cervical lymphadenopathy with a two-week duration and odynophagia. Bacterial tonsillitis was presumably diagnosed, and appropriate antibiotic therapy was prescribed. Two weeks later, the patient attended a same-day appointment at the healthcare center due to maintenance of left lateral cervical lymphadenopathy (2 cm) and weight loss of approximately 6 kg in a month. Laboratory tests were requested, which, when evaluated in a scheduled consultation two weeks later, showed no relevant alterations. In that same consultation, however, submandibular lymphadenopathies and a left supraclavicular one were also noted, all non-painful, with elastic consistency and mobile. A cervical ultrasound was requested in this context, which revealed lymphadenopathies with reactive characteristics. The patient was then referred for a biopsy, which he refused. He lost subsequent follow-up due to missed appointments.

2017 to 2020

For three years, the patient was sporadically observed in same-day consultations with multiple professionals for various reasons, including the persistence of cervical and axillary lymphadenopathies and intermittent complaints of retrosternal pain and a sensation of pressure in the lower throat, without clear association with effort, position, or swallowing, and sometimes with spontaneous resolution or partial improvement with non-steroidal anti-inflammatory drugs (NSAIDs). He underwent an electrocardiogram (ECG), which was normal, laboratory tests, and a new ultrasound without significant changes compared to previous examinations.

August 2021

The patient was seen in a new same-day appointment for pleuritic chest pain on the right side of his chest since the previous day, with worsening on deep inspiration, but without dyspnea. He also reported previous episodes of precordial pain lasting seconds, without a relationship with effort, trauma, or anxiety. Physical examination revealed local tenderness on palpation. He underwent chest radiography and ECG, both of which were unremarkable. When reassessed, a musculoskeletal etiology was considered, and symptomatic therapy with NSAIDs was instituted, with partial and transient improvement. He did not attend the next reassessment with his family doctor.

July to October 2023

He attended a new same-day consultation for pharyngeal discomfort described as a stuck fish bone and sensation of heartbeat at the level of the lower throat, without fever, cough, or other respiratory symptoms, having started these symptoms after vocal effort while attending a football match. He reported increased tobacco consumption as well. The patient returned twice the following month due to symptom maintenance. The first time was due to a post-nasal drip noted on physical examination; he was medicated with a nasal corticosteroid. The second time was due to the observation of a sub-centimeter papular lesion in the oropharynx and history of human papillomavirus (HPV) infection in his partner; he was referred to a sexually transmitted infections consultation, which he did not attend. Between September and October, he was observed in three other consultations, including one scheduled appointment, due to the persistence of odynophagia. One course of antibiotics for possible tonsillitis was attempted, without therapeutic success. Nevertheless, during this period, he began to associate this pharyngeal discomfort as a radiation of chest pain with characteristics similar to previous episodes, namely, of a stabbing type, with a duration of minutes to hours, aggravated by deep inspiration, without relationship to effort, anxiety, trauma, food intake, or position. No associated respiratory complaints or palpitations. On physical examination, he was normotensive with good peripheral saturation, and no relevant alterations were noted. Cycles of symptomatic medication with NSAIDs and proton pump inhibitors (PPIs) were prescribed and completed, with partial improvement. He underwent Holter monitoring and echocardiogram, both of which were unremarkable. The patient was sent to the emergency department due to sudden symptom worsening and was later discharged due to the absence of an obvious cause of pain and severity criteria.

November 2023

**Figure 1:**
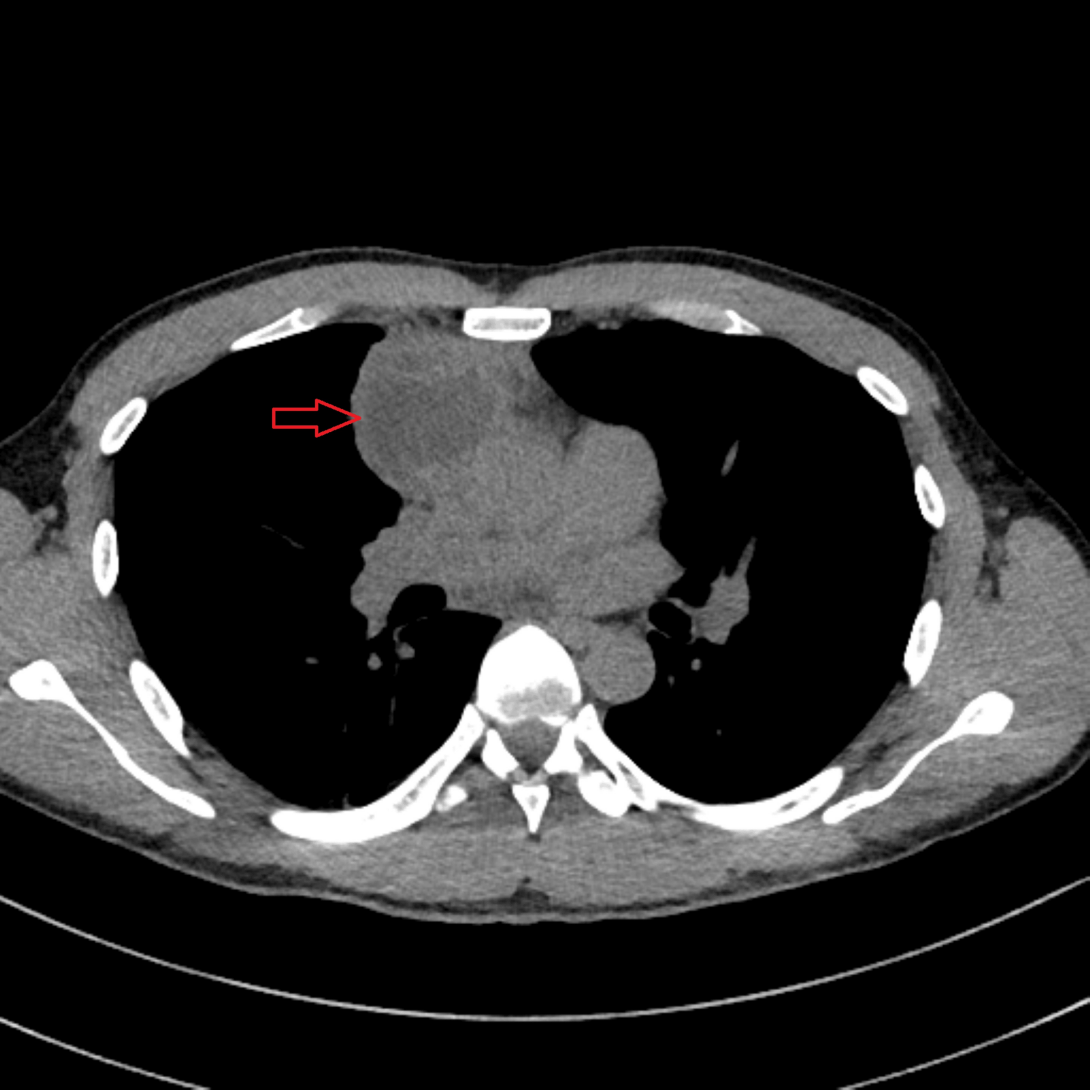
Axial CT scan image exhibiting an anterior mediastinal mass, with central areas of liquid density, and its relationship with surrounding structures

**Figure 2:**
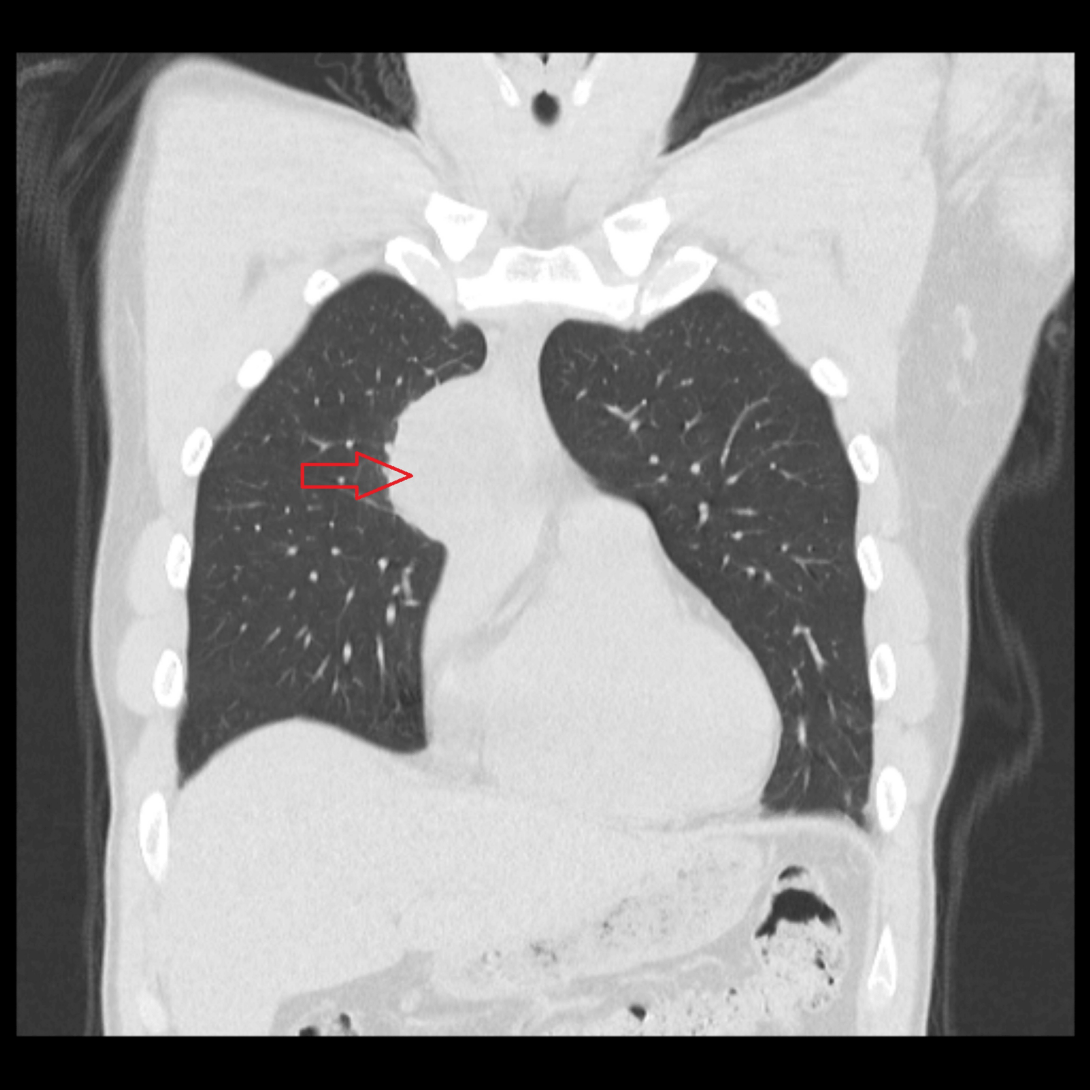
Coronal CT scan image exhibiting an anterior mediastinal mass and its relationship with surrounding structures

**Figure 3:**
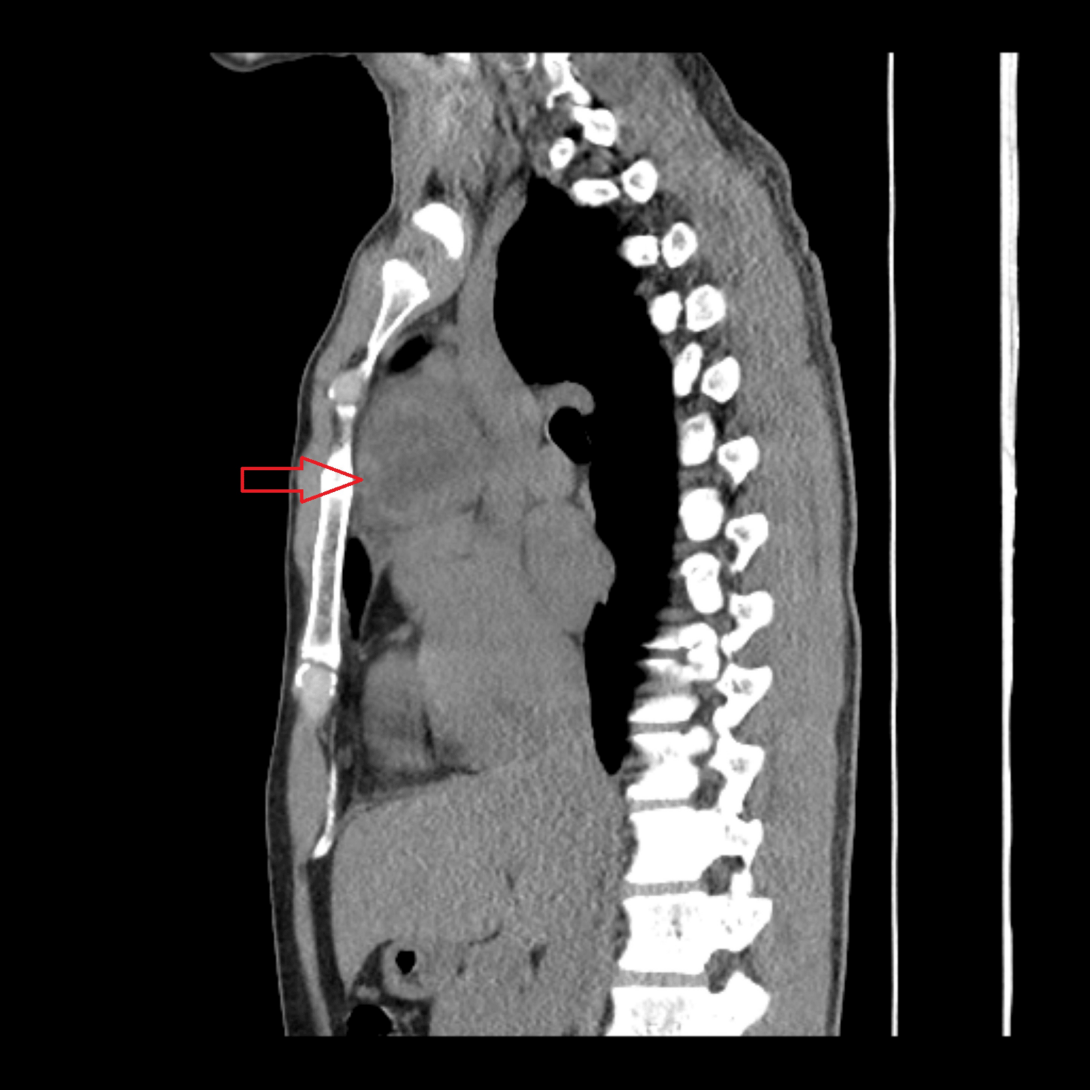
Sagittal CT scan image exhibiting an anterior mediastinal mass and its relationship with surrounding structures

Due to symptom worsening, a thoraco-abdominopelvic computed tomography (CT) scan was requested in a scheduled consultation, which reported a rounded mass estimated at 54 x 49 x 47 mm, with poorly defined contours, with central areas of liquid density, probably necrotic, in the prevascular anterior mediastinum. A cleavage plane with the ascending aorta was noted. Lymph node hypertrophy with a 9 mm short axis was observed, possibly translating to locoregional dissemination (Figures 1-3). Due to suspected malignancy, the patient was then referred to hospital care for continuation of care.

December 2023

Transthoracic needle aspiration biopsy (TNAB) was performed, with findings compatible with mature teratoma.

February 2024

Excision of the anterior mediastinal mass by video-assisted thoracoscopic surgery (VATS) in a hospital setting was performed. Intraoperatively, a hard mass of approximately 6 cm was found adherent to the pericardium and middle lobe, intimately related to the right phrenic nerve and brachiocephalic venous trunk. En bloc resection was performed, including the involved pericardium and atypical middle lobe resection.

The patient was discharged, clinically well, with complete resolution of symptoms. The pathology report stated the mass was a mature cystic teratoma, with areas of necrosis and free margins.

## Discussion

This case illustrates the challenges and complexity that follow-up and clinical reasoning in family medicine can pose.

The initial presentation in 2015 with cervical lymphadenopathy and odynophagia, although compatible with acute tonsillitis, evolved atypically with weight loss and persistence of lymphadenopathies. The identification of supraclavicular lymphadenopathy, recognized as often associated with thoracic or abdominal malignant pathology [2], warranted an appropriately motivated referral for specialized biopsy. The patient's refusal of the procedure and subsequent losses to follow-up constituted significant obstacles to the final diagnosis.

The intermittent complaints of chest pain and pharyngeal discomfort sensation, without a constant pattern or clear relationship with effort, food, position, trauma, or stress, represented a considerable diagnostic challenge. The initial absence of relevant alterations in complementary examinations, namely, laboratory tests, chest radiography, ECG, Holter monitoring, and echocardiogram, could suggest a benign etiology. However, the maintenance of lymphadenopathies and symptom persistence justified continuous reassessment. This follow-up, however, was complicated by the alternation of professionals who observed the patient in the context of same-day consultations and typically focused on acute or acutely worsened problems, which hindered the continuity of care usually provided by scheduled consultations with the family team.

The symptomatic worsening in 2023 motivated the performance of a thoraco-abdominopelvic CT scan, which revealed a 54×49×47 mm anterior mediastinal mass with adjacent lymphadenopathy, raising suspicion of malignancy and reinforcing the need for urgent referral. TNAB was performed, which confirmed the histological diagnosis of mature teratoma. The intraoperative findings, namely, involvement of the pericardium and proximity to the phrenic nerve and brachiocephalic venous trunk, may explain the variability of clinical presentation and its evolution throughout the case.

The eight-year interval between the first evaluation and definitive diagnosis, although multifactorial, was significantly influenced by the patient's irregular adherence to follow-up and the proposed investigation plan.

Although most chest pains and lymphadenopathies seen in primary health care are of benign origin, symptom persistence and/or presence of warning signs should motivate reassessment and investigation until even their rarest causes can be excluded. Mature teratomas generally present a favorable prognosis when completely resected, with low recurrence rates [6,7]. Although this diagnosis is associated with a positive outcome, it constitutes a pertinent opportunity for reflection on strategies that may minimize diagnostic delays in similar contexts.

This report underlines that, when faced with a myriad of possible causes for common symptoms, their persistence or the existence of warning signs should motivate systematic reassessment, which remains essential for the identification of potentially rare, serious, and clinically significant diagnoses.

## Conclusions

This case of mediastinal teratoma diagnosed after eight years of intermittent symptoms and follow-up demonstrates the fundamental importance of continuity of care in family medicine. The persistence of cervical lymphadenopathy and the development of atypical chest pain required longitudinal integration of clinical findings across multiple encounters. Despite challenges including patient non-adherence and consultation discontinuity, systematic reassessment ultimately led to the diagnosis of a rare but treatable condition. Family physicians must maintain vigilance for persistent symptoms and warning signs, even when initial investigations are unremarkable. This case emphasizes that comprehensive, patient-centered care and temporal integration of clinical data remain cornerstone principles of family medicine practice, essential for identifying uncommon diagnoses that may initially present with common symptoms.
